# t-DARPP regulates phosphatidylinositol-3-kinase-dependent cell growth in breast cancer

**DOI:** 10.1186/1476-4598-9-240

**Published:** 2010-09-13

**Authors:** Bhavatarini Vangamudi, Dun-Fa Peng, Qiuyin Cai, Wael El-Rifai, Wei Zheng, Abbes Belkhiri

**Affiliations:** 1 Department of Surgery, Vanderbilt University Medical Center, Nashville, Tennessee, USA; 2 Vanderbilt Epidemiology Center, Department of Medicine, Vanderbilt University Medical Center, Nashville, Tennessee, USA

## Abstract

**Background:**

Recent reports have shown that t-DARPP (truncated isoform of DARPP-32) can mediate trastuzumab resistance in breast cancer cell models. In this study, we evaluated expression of t-DARPP in human primary breast tumors, and investigated the role of t-DARPP in regulating growth and proliferation in breast cancer cells.

**Results:**

Quantitative real time RT-PCR analysis using primers specific for t-DARPP demonstrated overexpression of t-DARPP in 36% of breast cancers (13/36) as opposed to absent to very low t-DARPP expression in normal breast tissue (p < 0.05). The mRNA overexpression of t-DARPP was overwhelmingly observed in ductal carcinomas, including invasive ductal carcinomas and intraductal carcinomas, rather than other types of breast cancers. The immunohistochemistry analysis of DARPP-32/t-DARPP protein(s) expression in breast cancer tissue microarray that contained 59 tumors and matched normal tissues when available indicated overexpression in 35.5% of primary breast tumors that were more frequent in invasive ductal carcinomas (43.7%; 21/48). In vitro studies showed that stable overexpression of t-DARPP in MCF-7 cells positively regulated proliferation and anchorage-dependent and -independent growth. Furthermore, this effect was concomitant with induction of phosphorylation of AKT^ser473 ^and its downstream target phospho^ser9 ^GSK3β, and increased Cyclin D1 and C-Myc protein levels. The knockdown of endogenous t-DARPP in HCC1569 cells led to a marked decrease in phosphorylation of AKTs^ser473 ^and GSK3β^ser9^. The use of PI3K inhibitor LY294002 or Akt siRNA abrogated the t-DARPP-mediated phosphorylation of AKT^ser473 ^and led to a significant reduction in cell growth.

**Conclusions:**

Our findings underscore the potential role of t-DARPP in regulating cell growth and proliferation through PI3 kinase-dependent mechanism.

## Background

Breast cancer is a leading cause of death among women worldwide [1]. Identification of novel molecular targets and studies of signaling pathways that drive breast cancer tumorigenesis hold the premise for improvement of our current limited preventive, diagnostic, and therapeutic capabilities to breast malignancies. There is clear evidence that dysregulation of the PI3K/AKT signaling plays a central role in the pathogenesis of breast cancer [2-4]. The PI3K/AKT signaling regulates fundamental cellular processes linked to tumorigenesis, including cell growth, survival with resistance to therapy [3,5,6]. Recent studies have implicated activation of the PI3K/AKT pathway in conferring resistance to conventional chemotherapy and several chemotherapeutic agents (5-fluorouracil, adriamycin, mitomycin C, and cisplatinum) on cancer cells [7]. Somatic gain-of-function mutations of *PIK3CA *are associated with an increased activation of PI3K in breast cancers [8-10]. Functional analyses have revealed that they increase enzymatic activity, induce AKT signaling, and promote growth factor-independent growth as well as increase cell invasion and metastasis [11-13].

PPP1R1B, also known as dopamine and cyclic AMP (c-AMP)-regulated phosphoprotein of Mr 32,000 (DARPP-32), mainly expressed in the brain, is involved in dopaminergic neurotransmission and is a key factor in the functioning of dopaminoceptive neurons [14]. A comprehensive molecular analysis involving physical mapping strategies of transcripts in the ERBB2 amplicon region (17q12) pointed to the importance of EST AA552509 as the critical target [15,16]. Further studies of this EST using cloning and 3' and 5' RACE (rapid extension of cDNA ends) identified two transcripts [16]. The first transcript matched to DARPP-32. The second transcript was a transcriptional splice variant of DARPP-32 that encodes a truncated protein isoform that was named t-DARPP. t-DARPP lacks the NH2-terminal protein phosphatase inhibitory domain of DARPP-32, which is critical for dopamine signaling function in the brain, and is frequently overexpressed in several common adenocarcinomas, such as those of the stomach, colon, ovary and prostate [17-22]. In a previous study, we have demonstrated that t-DARPP can mediate the therapeutic resistance to trastuzumab through activation of the AKT pathway in breast cancer cells [23]. In this report, we have evaluated t-DARPP expression in human primary breast tumors, and investigated the role of t-DARPP in regulating cell growth and proliferation in breast cancer cells.

## Results

### Overexpression of t-DARPP in primary breast tumors

**Figure 1:**
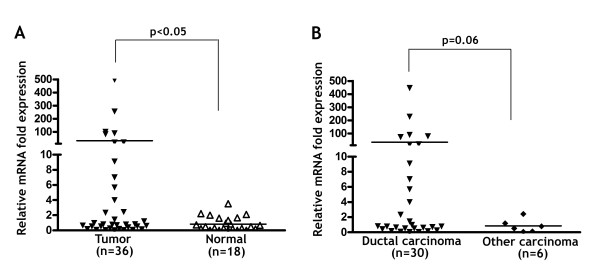
**t-DARPP is overexpressed in primary breast tumors**. **A**, normal adjacent (n = 18) and tumor (n = 36) breast tissue samples were analyzed by quantitative real time RT-PCR for t-DARPP mRNA expression. RT-PCR results were normalized with the internal control genes β-actin and *HPRT1*. The results were plotted as a ratio of normalized t-DARPP mRNA expression by the average of normalized t-DARPP mRNA expression of normal samples. Overexpression of t-DARPP was detected in 36% of breast tumors relative to normal tissue samples (p < 0.05). **B**, the overexpression of t-DARPP was overwhelmingly observed in ductal carcinomas (p = 0.06), including invasive ductal carcinomas and intraductal carcinomas, as opposed to other types of breast cancers. The other types of breast cancers include 1 invasive lobular carcinoma, 1 mucinous carcinoma, 1 apocrine carcinoma and 3 undifferentiated carcinomas. Horizontal bars indicate mean values. Mann Whitney test was used for statistical analysis in A and B.

We evaluated the mRNA expression of *t-DARPP *in 36 mRNA samples from primary breast tumors by quantitative real-time PCR using primers that can specifically detect *t-DARPP *unique sequence of the 5' UTR of exon 1. In addition, we analyzed 18 normal adjacent breast tissue samples for t-DARPP mRNA expression. The results were normalized to *HPRT1 *as a stable reference gene for quantitative real-time PCR. We detected overexpression of t-DARPP in 36% of breast cancers relative to normal breast tissues (p < 0.05) (Figure 1A). The mRNA expression of *t-DARPP *was absent to very low in normal breast tissue samples (Figure 1A). Based on the histological tumor type information, we found that the mRNA overexpression of t-DARPP was overwhelmingly observed in ductal carcinomas, including invasive ductal carcinomas and intraductal carcinomas, rather than other types of breast cancers (p = 0.06) (Figure 1B). The other types of breast cancers included 1 invasive lobular carcinoma, 1 mucinous carcinoma, 1 apocrine carcinoma, and 3 undifferentiated carcinomas.

We have attempted to evaluate the protein expression of t-DARPP in primary breast tumors by Immunohistochemistry analysis (IHC). As a specific antibody that exclusively detects t-DARPP was not available, we initially used the C-terminal DARPP-32 antibody (H-62; Santa Cruz Biotechnology), which detects both t-DARPP and DARPP-32 proteins, for immunohistochemical staining on tissue microarrays containing 59 primary breast tumor samples (FULL MOON Biosystems & AccuMax Array). IHC analysis of DARPP-32/t-DARPP proteins in breast tumor tissue samples indicated expression in both nucleus and cytoplasm with nuclear staining frequently stronger than cytoplasm staining [Additional file 1: Supplemental Figure S1]. Overall, our results indicated that high DARPP-32/t-DARPP expression scores (3+) were found in 35.5% of tumor samples as opposed to only 4% of normal tissue samples [Additional file 2: Supplemental Table S1]. The difference in DARPP-32/t-DARPP expression between normal tissue and tumor samples was statistically significant (p < 0.01). Based on the clinical information provided with the tissue microarrays, we found a statistically significant difference (p = 0.022) between DARPP-32/t-DARPP expression scores and histological tumor types (invasive ductal carcinoma or other). Our results indicated that 21 (43.7%) out of 48 invasive ductal carcinoma samples exhibited high (3+) DARPP-32/t-DARPP expression, the other histological carcinomas displayed absent or much lower expression [Additional file 2: Supplemental Table S1].

In an attempt to discriminate between the protein expression of DARPP-32 and t-DARPP, we performed immunohistochemical staining on duplicate TMA slides containing 19 primary breast tumor samples (AccuMax Array). We used the C-terminal DARPP-32 antibody (H-62; Santa Cruz Biotechnology), which detects both t-DARPP and DARPP-32 proteins, and the N-terminal DARPP-32 monoclonal antibody (EP720Y; abcam), which exclusively detects DARPP-32 protein. The results indicated that the immunostaining with C-terminal DARPP-32 antibody was relatively higher in 8 (42.1%) tumor samples than N-terminal DARPP-32 antibody. In contrast, only one (5.2%) tumor sample showed more immunostaining with N-DARPP-32 antibody [Additional file 3: Supplemental Table S2]. In fact, among the 8 tumor samples showing higher C-DARPP-32 IHC scores, 4 samples were completely negative for N-DARPP-32 immunostaining. These results suggested that protein expression of t-DARPP was higher than DARPP-32 in these analyzed primary breast tumor samples.

### Ectopically expressed t-DARPP promotes anchorage-dependent and anchorage-independent cell growth

**Figure 2:**
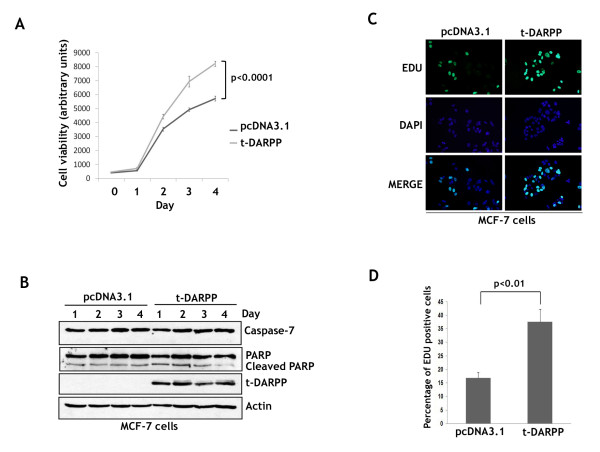
**t-DARPP ectopic overexpression promotes breast cancer cell growth and proliferation**. **A**, MCF-7 cells were stably transfected with t-DARPP or pcDNA3.1 empty vector control plasmids. The cells (2 × 10^3 ^cells per well) were seeded onto 96-well culture plates and grown for 1, 2, 3, and 4 days and then subjected to Cell Titer-Glo Luminiscent Cell Viability Assay. Growth rate of MCF-7 cells stably expressing t-DARPP (grey color) was significantly higher (p < 0.0001) than empty vector control cells (black color). **B**, the cells (2 × 10^5 ^cells per well) were seeded onto 6-well culture plates and grown for 1, 2, 3, and 4 days. Protein extracts from these cell cultures were subjected to western blot analysis of t-DARPP, Caspase-7, PARP, and β-actin. The results indicated that t-DARPP-induced cell growth over 4 days was not affected by apoptosis as shown by Caspase-7 and PARP. **C**, MCF-7/pcDNA3.1 and MCF-7/t-DARPP cells were grown for 48 h then subjected to the Click-iT EdU proliferation Assay. Proliferating cells were depicted by EdU positive staining (green fluorescence) and all the cells were stained with DAPI (nuclear blue fluorescence). **D**, a summary of the results showing the percent of proliferating cells. Cell proliferation of t-DARPP-expressing cells (37%) was significantly higher than control cells (16%, p < 0.01). Results are representative of at least three experiments and shown as the mean ± SD. Significance of difference was calculated using Student's t test.

To investigate the effect of ectopic t-DARPP overexpression on cell growth, we developed a cell model by stably expressing t-DARPP or pcDNA3.1 empty vector in MCF-7 cells, which are negative for endogenous t-DARPP expression. The cells were grown for several days and then subjected to Cell Titer-Glo Luminiscent Cell Viability assay. Our results confirmed that t-DARPP ectopic expression in MCF-7 cells significantly enhanced cell growth starting at day 1 and reaching approximately 44% growth increase relative to control cells at day 4 (p < 0.0001) (Figure 2A). To confirm that t-DARPP-induced cell growth results were not affected by apoptosis as a result of growth conditions, we subjected protein extracts from a similar cell growth experiment to western blot analysis of Caspase-7, PARP, and t-DARPP. Indeed, the results showed that the experimental growth conditions did not induce significant apoptosis as indicated by Caspase-7 and PARP protein levels (Figure 2B). To ascertain that t-DARPP-induced cell growth was due to increased proliferation, we subjected MCF-7/pcDNA3.1 and MCF-7/t-DARPP cells that were grown for 48 h to the Click-iT EdU cell proliferation assay (Invitrogen). The proliferating cells were positive for EdU signal as depicted by green fluorescence, and all the proliferating and non-proliferating cells were counterstained with DAPI (blue nuclear fluorescence) (Figure 2C). The results indicated that cell proliferation of t-DARPP-expressing cells (37%) was significantly higher than control cells (16%, p < 0.01) (Figure 2D). These findings clearly showed that t-DARPP expression promotes cell proliferation in breast cancer cells.

**Figure 3:**
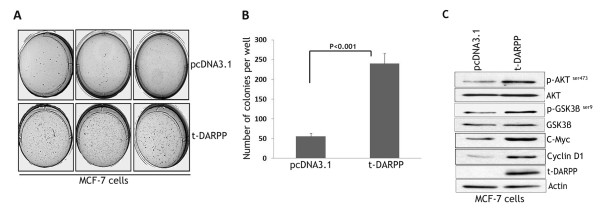
**t-DARPP expression enhances anchorage-independent cell growth in soft agar**. **A**, MCF-7 cells stably expressing t-DARPP or pcDNA3.1 empty vector were seeded in soft agar onto 6-well tissue culture plates (10^5 ^cells per well). The cells were grown for one month to allow for anchorage-independent colony formation. **B**, Quantification of colony formation indicated that MCF-7/t-DARPP cells produced markedly higher number of colonies than control cells (>4-fold increase, p < 0.001). **C**, Protein extracts from MCF-7/t-DARPP and MCF-7/pcDNA3.1 cells were analyzed by western blot for t-DARPP, p-AKT (S473), AKT, p-GSK3β (S9), GSK3β, C-Myc, Cyclin D1, and β-actin. t-DARPP expression directly correlated with increased phospho-AKT^ser473^, phospho-GSK3β^ser9^, C-Myc, and Cyclin D1 protein levels. Results are representative of at least three experiments and shown as the mean ± SD. Significance of difference was calculated using Student's t test analysis.

Anchorage-independent growth capability is one of the hallmarks of oncogenically transformed cells. Furthermore, increased anchorage-independent growth may potentially indicate enhanced cell tumorigenicity in vivo. To investigate the role of t-DARPP in regulating anchorage-independent growth in breast cancer cells, we examined colony formation on soft agar using the MCF-7/t-DARPP cell model described above. The results showed that t-DARPP-expressing cells produced markedly higher number of colonies (>4-fold increase, p < 0.001) than control cells (Figure 3A &3B). This enhanced anchorage-independent growth capability of t-DARPP-expressing MCF-7 cells was associated with increased p-AKT^ser473 ^and p-GSK3β^ser9 ^protein levels as compared with control cells (Figure 3C). In addition, we examined the expression of β-catenin downstream signaling effectors Cyclin D1 and C-Myc, and found a significant increase of their protein levels in t-DARPP-expressing cells as compared with control cells (Figure 3C). These results suggest that t-DARPP regulates AKT signaling in vitro.

### Endogenous t-DARPP expression correlates with activation of AKT signaling pathway in breast cancer cell lines

**Figure 4:**
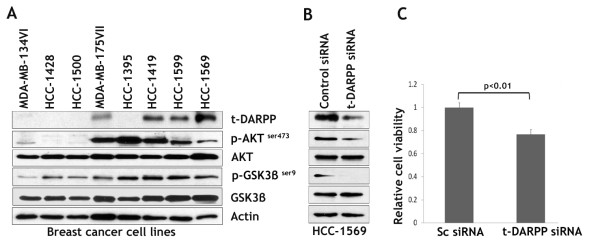
**t-DARPP expression directly correlates with activation of AKT pathway in breast cancer cell lines, and knockdown of endogenous t-DARPP suppresses cell growth**. **A**, Protein extracts from a panel of 8 breast cancer cell lines were subjected to western blot analysis of t-DARPP, p-AKT^ser473^, AKT, p-GSK3β^ser9^, GSK3β, and β-actin. 10 μg of total protein per lane were resolved on 10% SDS-PAGE and transferred onto Hybond-P PVDF membranes for immunodetection with the corresponding specific antibodies. **B&C**, HCC-1569 cells were transfected with control scrambled siRNA or t-DARPP siRNA oligonucleotides and grown for 48 h. Protein extracts were subjected to western blot analysis of t-DARPP, p-AKT^ser473^, AKT, p-GSK3β^ser9^, and GSK3β (panel B). Knockdown of t-DARPP in HCC-1569 cells led to down-regulation of the AKT signaling pathway as indicated by a significant decrease of p-AKT^ser473 ^and p-GSK3β^ser9 ^protein levels. The cells were subjected to Cell-Titer-Glo Luminescent Cell Viability Assay. Overall, cell growth was significantly lower in HCC-1569 cells transfected with t-DARPP siRNA than control cells (panel C). Results are representative of three experiments and shown as the mean ± SD. Significance of difference was calculated using Student's t test analysis.

As we have shown that ectopic t-DARPP expression was associated with activation of the AKT pathway in MCF-7 cell model (Figure 3C), we sought to determine if this association was established in a panel of 8 additional breast cancer cell lines. We have shown t-DARPP protein expression in multiple breast cancer cell lines by western blot analysis as indicated by a specific 28 kDa protein band (Figure 4A). Overall, our results indicated that all the t-DARPP-expressing cells (MDA-MB-175VII, HCC-1419, HCC-1599, and HCC-1569) exhibited a significantly higher p-AKT^ser473 ^and p-GSK3β^ser9 ^protein levels as compared with t-DARPP-non-expressing cell lines (MDA-MB-134VI, HCC-1428, and HCC-1500) (Figure 4A). In contrast, HCC-1395 cells were the exception as they are negative for t-DARPP expression but showed high p-AKT^ser473 ^and p-GSK3β^ser9 ^protein levels (Figure 4A). Interestingly, HCC-1395 cells were reported to be negative for expression of PTEN, a negative regulator of PI3K/AKT pathway, as a result of a homozygous *PTEN *deletion-frame-shift mutation (Sanger Institute, UK), thus explaining the activation of the AKT signaling pathway. Taken together, our results showed a direct correlation between t-DARPP expression and activation of the AKT pathway in the tested breast cancer cell lines.

To investigate the role of endogenous t-DARPP in regulating the AKT pathway and cell growth, we knocked down t-DARPP expression with siRNA in HCC-1569 cells and then evaluated the AKT signaling effectors by western blot analysis. In parallel, we determined the effect of t-DARPP knockdown on cell growth using the Cell Titer-Glo Luminescent Cell Viability Assay. The knockdown of endogenous t-DARPP resulted in down-regulation of the AKT pathway as indicated by decreased levels of p-AKT^ser473 ^and p-GSK3β^ser9 ^proteins in HCC-1569 cells as compared with control cells (Figure 4B). In addition, the results showed that t-DARPP knockdown led to a marked decrease (20%) in cell viability in HCC-1569 cells as compared with cells transfected with control siRNA (p < 0.01) (Figure 4C). Together, these findings suggested that t-DARPP-dependent cell growth implicated regulation of the AKT signaling pathway in breast cancer cells.

### t-DARPP induces cell growth through PI3 kinase-dependent mechanism

**Figure 5:**
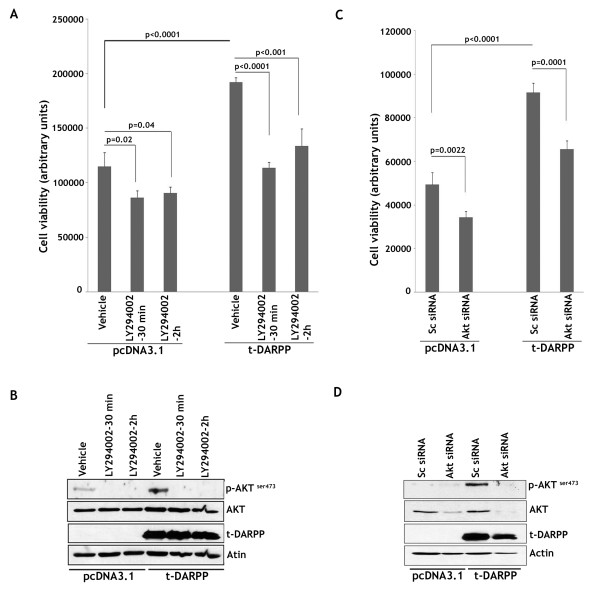
**t-DARPP-induced cell growth is dependent on PI3K/AKT signaling pathway**. **A**, MCF-7 cells stably expressing t-DARPP or pcDNA3.1 vector were treated with either vehicle or 40 μM of LY294002 for 30 min or 2 h. The cells were washed with PBS and grown for 24 h, and then subjected to Cell-Titer-Glo Luminescent Cell Viability Assay. The results indicated a significant increase of cell growth in t-DARPP-expressing cells relative to control cells after treatment with vehicle. This growth increase was almost completely abrogated after treatment with LY294002. **B**, MCF-7/t-DARPP and MCF-7/pcDNA3.1 cells were treated with vehicle and 40 μM of LY294002 for 30 min or 2 h, and then grown for 24 h. Protein extracts were subjected to western blot analysis of t-DARPP, p-AKT^ser473^, and AKT. The results showed that p-AKT^ser473 ^protein level was significantly higher in t-DARPP-expressing cells than control cells, and treatment with LY294002 blocked phosphorylation of AKT^ser473 ^in all cells. **C**, MCF-7/t-DARPP and MCF-7/pcDNA3.1 cells were transfected with scrambled siRNA or Akt siRNA and grown for 48 h. The cells were then subjected to Cell-Titer-Glo Luminescent Cell Viability Assay. The results indicated a significant increase of cell growth in t-DARPP-expressing cells relative to control cells after transfection with scrambled siRNA. This growth increase was partially abrogated after transfection with Akt siRNA. **D**, MCF-7/t-DARPP and MCF-7/pcDNA3.1 cells were transfected with scrambled siRNA or Akt siRNA and cultured for 48 h. Protein extracts were analyzed by Western blot for p-AKT^ser473^, AKT, t-DARPP, and β-actin. Results are representative of four experiments and shown as the mean ± SD. Significance of difference was calculated using Student's t test analysis.

Our results demonstrated that t-DARPP-regulated cell growth directly correlated with the activity of AKT pathway as indicated by protein levels of p-AKT^ser473 ^and its downstream substrate p-GSK3β^ser9 ^in breast cancer cells expressing ectopic or endogenous t-DARPP protein (Figures 3 &4). To determine if t-DARPP-induced cell growth was dependent on PI3K/AKT pathway, we treated MCF-7/t-DARPP and MCF-7/pcDNA3.1 cells with 40 μM of the PI3K specific pharmacologic inhibitor LY294002 (Cell Signaling) or vehicle (DMSO) for 30 min or 2 h, and the cells were grown for an additional 24 h. The cells were then subjected to Cell-Titer-Glo Luminescent Cell Viability Assay to measure cell growth. The results showed a significant increase of growth (approximately 67%) in t-DARPP-expressing cells relative to control cells after treatment with vehicle (p < 0.0001) (Figure 5A). In contrast, treatment with LY294002 inhibitor led to a significant average growth decrease of 36% (p < 0.001) relative to treatment with vehicle in t-DARPP-expressing cells (Figure 5A). In fact, the growth decrease (36%) resulting from LY294002 treatment in t-DARPP-expressing cells approximately accounted for the growth increase in t-DARPP-expressing cells relative to control cells after treatment with vehicle. Interestingly, treatment with LY294002 led to a growth decrease of approximately 24% relative to vehicle in control cells as opposed to 36% in t-DARPP-expressing cells (Figure 5A). This indicated that t-DARPP-expressing cells were more sensitive to LY294002, and therefore to inhibition of PI3K/AKT pathway, than control cells. Together, our results showed that treatment with LY294002 inhibitor abrogated t-DARPP-induced cell growth in MCF-7 cells. In addition, our western blot analysis results indicated that, as expected, p-AKT^ser473 ^protein level was significantly higher in t-DARPP-expressing cells as compared with control cells following treatment with vehicle. In contrast, treatment with LY294002 inhibitor completely blocked phosphorylation of AKT in all cells (Figure 5B). These results clearly demonstrated that t-DARPP-induced growth in MCF-7 cells was dependent on activation of PI3K/AKT pathway as inhibition of PI3K with LY294002 led to almost complete abrogation of t-DARPP function. To further confirm that t-DARPP-induced cell growth was dependent on PI3K/AKT pathway, we transfected MCF-7/t-DARPP and MCF-7/pcDNA3.1 cells with Akt siRNA or control scrambled siRNA oligonucleotides, and the cells were cultured for 48 h. The cells were then subjected to Cell-Titer-Glo Luminescent Cell Viability Assay to measure cell growth. The results indicated a significant increase of growth (approximately 85%) in t-DARPP-expressing cells relative to control cells transfected with control scrambled siRNA (p < 0.0001) (Figure 5C). In contrast, transfection with Akt siRNA led to a significant growth decrease of 28.5% (p = 0.0001) relative to transfection with control scrambled siRNA in t-DARPP-expressing cells (Figure 5C). This growth decrease resulting from AKT knockdown indicated a partial abrogation of the growth increase of t-DARPP-expressing cells relative to control cells. Our western blot analysis results clearly indicated knockdown of AKT and p-AKT^ser473 ^after transfecting cells with Akt siRNA oligonucleotide (Figure 5D). In summary, these results confirmed that t-DARPP-induced growth in MCF-7 cells was dependent on activation of PI3K/AKT pathway as inhibition of PI3K with LY294002 or knocking down AKT with Akt siRNA led to abrogation of t-DARPP cell growth promoting function.

## Discussion

In this study, we have demonstrated that t-DARPP regulates cell growth and proliferation through activation of PI3K/AKT pathway in human breast cancer cells. To evaluate the specific expression of t-DARPP in breast tissue samples, we employed real-time RT-PCR using primers that can specifically detect *t-DARPP *unique sequence of the 5'UTR region of exon 1. We have shown for the first time that *t-DARPP *was overexpressed at the mRNA level in 36% of primary breast tumors relative to normal breast tissues (p < 0.05). In addition, we demonstrated that the overwhelming majority of samples that overexpressed t-DARPP were ductal carcinomas as opposed to other carcinomas (p = 0.06). Although statistically not significant (p = 0.06), the difference in t-DARPP expression level between ductal carcinomas and other types of carcinomas indicated a trend that could be confirmed by analyzing additional tumor samples.

As an exclusively specific antibody for t-DARPP was not available, we evaluated the expression of DARPP-32/t-DARPP protein(s) in primary breast tumors by IHC analysis using C-terminal DARPP-32 antibody. We showed overexpression of DARPP-32/t-DARPP protein(s) in 35.5% of tumors. While our finding is consistent with a recent report [24], we demonstrated that high level of expression of DARPP-32/t-DARPP protein(s) (3+) was exclusively seen in invasive ductal carcinomas (IDC). This is an important observation since these tumors are characterized by a poor prognosis [25,26]. IDC is a common type of breast cancer, which has the potential to invade lymph nodes and blood systems, and eventually metastasize to other parts of the body, suggesting a possible role of DARPP-32/t-DARPP protein(s) in tumor progression [27-29]. In an attempt to discriminate between DARPP-32 and t-DARPP protein expression, we performed IHC analysis on duplicate breast tumor TMA with C-terminal DARPP-32 antibody, which recognizes both DARPP-32 and t-DARPP proteins, and N-terminal DARPP-32 antibody, which exclusively detects DARPP-32 protein. The IHC results indicated that 42.1% of tumors stained with both antibodies. However, the immunostaining with C-terminal DARPP-32 antibody was relatively higher than N-terminal DARPP-32 antibody. Half of these tumors were exclusively positive for immunostaining with C-terminal DARPP-32 antibody. These results suggested that t-DARPP protein expression was predominant in breast tumors as opposed to expression of DARPP-32 protein. Therefore, the immunostaining with C-terminal DARPP-32 antibody would mostly indicate the expression of t-DARPP protein. Taken together, our results showed that the frequency of t-DARPP mRNA overexpression (36%) was comparable to that of high DARPP-32/t-DARPP protein(s) expression (35.5%) in primary breast tumors.

In this current study, we have clearly demonstrated that endogenous and ectopically expressed t-DARPP exhibited a novel biological function by enhancing cell growth and proliferation in breast cancer cells. We have confirmed that t-DARPP-induced cell growth was not significantly affected by apoptosis in our in vitro MCF-7 cell model as indicated by Caspase-7 and PARP proteins. Generally, in absence of chemotherapeutics, cancer cells have little apoptosis. Therefore, the predominant function of t-DARRP in this case is to induce cell growth. In addition, we have investigated the role of t-DARPP in regulating anchorage-independent cell growth, which is one of the hallmarks of oncogenically transformed cells, and enhanced anchorage-independent growth capability may potentially increase cell tumorigenicity. Indeed, we have shown that t-DARPP expression significantly increased anchorage-independent growth capability of MCF-7 cells.

As part of an attempt to identify the signaling pathway that could be implicated in the regulation of t-DARPP-induced cell growth, we confirmed a direct correlation between expression of t-DARPP and activation of AKT pathway as indicated by increased phospho-AKT^ser473 ^and phospho-GSK3β^ser9 ^protein levels in multiple breast cancer cell lines. We have also shown that t-DARPP-induced cell growth and proliferation were associated with phosphorylation of GSK3β^ser9 ^and increased growth signaling effectors C-Myc and Cyclin D1 protein levels. The regulation of Cyclin D1 and C-Myc can be mediated by β-catenin as well as other signaling mechanisms. Future studies are required to fully elucidate the mechanism by which t-DARPP regulates *C-Myc *and *Cyclin D1 *genes.

Several reports indicated that dysregulation of the PI3K/AKT pathway plays a major role in the pathogenesis of breast cancer [2-4]. The PI3K/AKT signaling regulates cellular processes associated with breast tumorigenesis, including survival and cell growth [2,3]. Recent reports suggested the role of t-DARPP in mediating trastuzumab resistance in vitro mainly through modulation of the AKT signaling pathway in breast cancer cells [23,24,30]. In this study, we demonstrated that t-DARPP-induced cell growth was regulated through PI3K/AKT-dependent mechanism as inhibition of PI3 kinase with LY294002 and knocking down AKT with siRNA significantly abrogated t-DARPP function. This indicates that t-DARPP signaling to AKT is likely upstream of PI3 Kinase. In addition, inhibition of PI3K with LY294002 led to a substantial decrease of protein levels C-Myc and Cyclin D1 in t-DARPP-expressing cells (data not shown). This suggests that t-DARPP-induced growth and proliferation is regulated by t-DARPP-dependent activation of AKT and potentially β-catenin signaling pathway.

In this report, we have shown that t-DARPP regulated AKT pathway in ERBB2-positive cells (HCC-1569) and ERBB2-negative cells (MCF-7). It is possible that t-DARPP is involved in regulation of RTKs or PTEN that are main drivers of PI3K/AKT activation [31-33]. Further studies are required to elucidate the potential molecular mechanisms by which t-DARPP regulates PI3K/AKT signaling in cancer cells. The t-DARPP-induced anchorage-dependent and -independent cell growth underscores the oncogenic properties of t-DARPP whose overexpression may play an important role in breast tumorigenesis. The results from our MCF-7 cell model suggest that evaluation of t-DARPP mRNA expression with quantitative real-time PCR in tumors may prove useful for clinical management of breast cancer patients. For instance, as we have shown that t-DARPP function was dependent on activation of PI3K/AKT signaling, targeting AKT pathway or RTKs in cancer patients who overexpress t-DARPP could significantly improve the treatment of breast cancer.

## Conclusions

Our findings highlight the important role of t-DARPP in breast tumorigenesis by regulation of cell growth and proliferation through PI3K/AKT-dependent mechanism. Further studies are necessary to explore the mechanism by which t-DARPP regulates the PI3K/AKT signaling pathway in breast cancer cells.

## Methods

### Cell lines and plasmids

The human breast cancer cell lines, SKBR-3, MCF-7, MDA-MB-134VI, HCC-1428, HCC-1500, MDA-MB-175VII, HCC-1395, HCC-1419, HCC-1599, and HCC-1569 cells were obtained from the American Type Culture Collection (ATCC, Rockville, MD). All cells were maintained in F12 medium supplemented with 10% fetal bovine serum and 2 mM L-glutamine. The expression plasmid for t-DARPP was prepared by PCR amplification of the full-length coding sequence of t-DARPP (accession no. AY070271) and cloned in-frame into pcDNA3.1 (Invitrogen Life Technologies). MCF-7 stably expressing t-DARPP or pcDNA3.1 empty vector were generated following standard protocols as described previously [34]. After selection with 500 μg/ml neomycin (G418) (Invitrogen Life Technologies), t-DARPP protein expression was evaluated by western blot analysis.

### Quantitative real-time PCR

Quantitative real-time PCR was performed in triplicate using an iCycler (Bio-Rad) with a threshold cycle number determined by use of iCycler software version 3.0. Single-stranded cDNA was synthesized from a total RNA amount of 1 μg using the Advantage Reverse Transcription-PCR kit (Clontech). mRNA specific primers for *t-DARPP*, β-actin, and *HPRT1 *were designed, and the results were normalized to *HPRT1 *as a stable reference gene for quantitative real-time PCR. All primer sequences are available upon request. The mRNA fold expression levels were calculated according to the formula 2^(RT-ET)^/2^(Rn-En)^, as described previously [16]. We used an arbitrary cutoff value of 2-fold as a minimum for any overexpression.

### Immunohistochemistry

Tissue microarrays (TMA) containing 59 de-identified, archival cases of breast tumors, including 25 adjacent "normal" breast tissue samples, were obtained from FULL MOON Biosystems and AccuMax Array. All the cases provided the information of the original pathology diagnosis. 5 μM TMA sections were used for IHC staining of DARPP-32/t-DARPP using rabbit polyclonal DARPP-32 antibody (Clone H-62; 1:200 dilution, Santa Cruz Biotechnology, Inc., Santa Cruz, CA), which specifically recognizes the C-terminal end of both DARPP-32 and t-DARPP proteins. A TMA containing 19 breast tumors (AccuMax Array) was used for IHC staining with the C-terminal DARPP-32 antibody (described above) and rabbit monoclonal DARPP-32 antibody (Clone EP720Y; 1:250 dilution, abcam Inc., Cambridge, MA), which exclusively recognizes the N-terminal end of DARPP-32 protein. De-waxing and rehydration by descending concentrations of ethanol was followed by antigen retrieval (20 minutes in a microwave, 450 W, 10 mM EDTA, pH 8.0). 10 minutes blocking was performed with 10% goat serum in PBS. The sections were incubated overnight with either C-terminal or N-terminal DARPP-32 primary antibody, followed by washing in PBS and incubation with anti-rabbit secondary antibody for 1 hour at room temperature. The Vectastain ABC-AP KIT (vector; Alexis, Gruenberg, Germany) was used as the chromogen substrate, and the specimens were counterstained with hematoxylin. Specificity of immunostaining was checked by replacing the primary antibody with non-immune serum. A known positive breast cancer tissue slide was used as a positive control. DARPP-32/t-DARPP expression level was estimated semi-quantitatively [35]. Cores with torn tissues were excluded from the analyses. Cores with no evidence of nuclear or cytoplasmic staining, or only rare scattered positive cells less than 3%, were recorded as negative. The overall intensity of staining was recorded as that for the core with the strongest intensity. Immunohistochemical results were evaluated for intensity and frequency of staining of nuclear and cytoplasmic components and the whole. The intensity of staining was graded as 0 (negative), 1 (weak), 2 (moderate), and 3 (strong). The frequency was graded from 0 to 4 by percentage of positive cells as follows: grade 0, <3%; grade 1, 3~25%; grade 2, 25~50%; grade 3, 50~75%; grade 4, more than 75%. The index score was the product of multiplication of the intensity and frequency grades, which was then pinned into a 4 point scale: index score 0 = product of 0, index score 1 = products 1 to 4, index score 2 = products 5 and 9, index score 3 = products 10 to 12.

### Immunoblot analysis

Protein extracts (10 to 20 μg/lane) were resolved on 10% SDS-PAGE and then transferred on Hybond-P PVDF membranes (Amersham Biosciences), and subjected to western blot analysis. Rabbit polyclonal DARPP-32 antibody (clone H-62; 1:1000 dilution), which recognizes the C-terminal end of t-DARPP protein, and mouse monoclonal C-Myc antibody (Clone 9E10; 1:500 dilution) were obtained from Santa Cruz Biotechnology, Inc., Santa Cruz, CA. Mouse monoclonal AKT antibody (Clone 40D4; 1:2000 dilution), which recognizes the C-terminal end of AKT protein; rabbit polyclonal phospho-AKT^ser473 ^antibody (1:1000 dilution); rabbit monoclonal GSK3β antibody (Clone 27C10; 1:1000 dilution); rabbit monoclonal phospho-GSK3β^ser9 ^antibody (Clone 5B3; 1:1000 dilution); rabbit polyclonal Cyclin D1 antibody (1:1000 dilution); and rabbit polyclonal β-actin antibody (1:1000 dilution) were obtained from Cell Signaling Technology, Inc., Danvers, MA. Horseradish peroxidase-conjugated anti-mouse (1:5000 dilution) and anti-rabbit (1:5000 dilution) secondary antibodies were purchased from Santa Cruz Biotechnology and Cell Signaling Technology, respectively. Immunoreactive protein bands were visualized by enhanced chemiluminescence (Pierce).

### Cell viability/growth assay

Cells (2 × 10^3 ^per well) were seeded onto a 96-well plate. The cell viability of these cells after knockdown of endogenous t-DARPP or ectopic expression of t-DARPP was determined using the Cell Titer-Glo Luminescent Cell Viability Assay kit (Promega) following the supplier's instructions.

### Cell proliferation assay

To measure cell proliferation, we have used the Click-iT EdU Assay (Invitrogen), which is an alternative to the BrdU assay. EdU (5-ethynyl-2'-deoxyuridine) is a nucleoside analog of thymidine and is incorporated into DNA during active DNA synthesis. Briefly, cells (15 × 10^3 ^per chamber) were seeded onto 8-chamber slides and incubated at 37°C in an atmosphere containing 5% CO_2 _for 48 h. For labeling cells with EdU, equal volume of 2 X EdU solutions was added to the cells, and incubated at 37°C/5% CO_2 _for 60 min. The cells were then fixed with 3.7% formaldehyde in PBS at room temperature for 15 min, followed by permeabilization with 0.5% Triton X-100 in PBS for 20 min at room temperature. In this assay, detection of the signal is based on a click reaction, a copper-catalyzed covalent reaction between the EdU and the Alexa Fluor 488 dye. Click-iT reaction cocktail was added to the cells and incubated for 30 min at room temperature, and protected from the light. Following removal of click-iT reaction cocktail, the cells were washed twice with 1 ml 3% BSA in PBS. Prior to examination with fluorescence microscopy, Vectashield mounting medium with 4',6-diamidino-2-phenylindole (DAPI) (Vector Laboratories, Inc.) was added to the cells. EdU-positive cells (20 random fields at 40X, >400 cells) were counted. The percentage of proliferation of control and t-DARPP-expressing cells was determined by counting EdU-positive cells (green fluorescence) versus total number of cells, indicated by nuclear DAPI staining (blue fluorescence).

### Gene expression knockdown with small interfering RNA

The HCC-1569 cells were transfected with control scrambled siRNA (sc-37007) or t-DARPP siRNA (sc-35173) using siRNA transfection reagent (sc-29528) and transfection medium (sc-36868) following the manufacturer's instructions (Santa Cruz Biotechnology). The MCF-7/pcDNA3.1 and MCF-7/t-DARPP cells were transfected with control scrambled siRNA (#6568; Cell Signaling Technology) or Akt siRNA I (#6211; Cell Signaling Technology) using RNAifectin transfection reagent (#G073; abm Applied Biological Materials) following the manufacturer's instructions.

### Soft agar colony formation assay

Bottom layers of 1 ml 1% noble agar (Difco) in F12 medium, supplemented with 10% FBS, were prepared in 6-well tissue culture plates. Cells (10^5 ^per well) were suspended in top layers of 1 ml 0.7% noble agar in F12 medium, supplemented with 10% FBS. Triplicate cultures were established for each cell line. After preparation of both bottom and top layers, the plates were placed in a 5% CO_2 _atmosphere humidified incubator at 37°C. Colonies (≥1 mm) were observed then counted following approximately 1 month of incubation.

### Statistical analysis

The results were expressed as the mean with SD. The parametric unpaired Student's t test was used to assess the difference of growth and proliferation between MCF-7/pcDNA3.1 and MCF-7/t-DARPP cells. The non-parametric Mann Whitney test was used to evaluate the difference of t-DARPP mRNA fold expression of tumors vs. normal tissue samples and ductal carcinomas vs. other types of carcinomas. The Fisher Exact test was used to assess the difference between DARPP-32/t-DARPP IHC index score and cases (normal vs. tumor), or histology (IDC vs. other). p < 0.05 was defined as the statistical significance.

## Abbreviations

HPRT1: hypoxanthine phosphoribosyltransferase; IDC: invasive ductal carcinoma; IHC: immunohistochemistry; PCR: polymerase chain reaction; PI3K: phosphatidylinositol-3-kinase; RTKs: receptor tyrosine kinases; TMA: tissue microarray; t-DARPP: truncated dopamine and cyclic-AMP-regulated phosphoprotein of Mr 32,000 (DARPP-32); 5'UTR: 5' untranslated region.

## Competing interests

The authors declare that they have no competing interests.

## Authors' contributions

BV was involved in planning and performing experiments related to western blot analysis and functional assays. DP carried out IHC analysis and contributed to writing the manuscript. QC and WZ were involved in the real-time PCR analysis of the primary breast tumors. WER participated in the writing and organization of the manuscript. AB participated in the design of the experiments and writing the manuscript, and supervised the work relevant to this report. All the authors read and approved the final manuscript.

## Supplementary Material

Additional file 1 **Supplemental Figure S1. t-DARPP expression in primary breast tumors**. A representative immunohistochemical staining for DARPP-32/t-DARPP of a breast tumor (upper panel) and a matched adjacent normal tissue (lower panel) samples from a tissue microarray with C-terminal DARPP-32 antibody. Note that the expression of DARPP-32/t-DARPP protein(s) is high in tumor tissue as opposed to very low to absent in adjacent normal tissue, as indicated by dark brown staining. Click here for file

Additional file 2 **Supplemental Table S1. Summary of DARPP-32/t-DARPP immunostaining from breast tumor microarrays**. DARPP-32/t-DARPP protein(s) expression was assessed by IHC staining on tissue microarrays containing 59 primary breast tumors and 25 adjacent normal breast tissue samples using C-terminal DARPP-32 antibody. Immunohistochemical results were evaluated for intensity and frequency of staining. The index score of staining was graded as 0 (negative), 1 (weak), 2 (moderate), and 3 (strong). The difference between DARPP-32/t-DARPP expression frequency and various parameters was assessed by Fisher Exact Test. IDC, invasive ductal carcinoma. Click here for file

Additional file 3 **Supplemental Table S2. Differential expression of DARPP-32 and t-DARPP in breast cancer tissues**. IHC analysis of duplicate tissue microarray slides containing 19 primary breast tumor samples. Immunohistochemical staining was performed with N-terminal DARPP-32 antibody (EP720Y; abcam) (N-DARPP-32), which exclusively detects DARPP-32 protein, and C-terminal DARPP-32 antibody (Clone H-62; Santa Cruz Biotechnology) (C-DARPP-32), which detects both DARPP-32 and t-DARPP proteins. Both antibodies produced similar strong and specific staining in positive cases. The results indicated that 8 (42.1%) tumors exhibited relatively higher t-DARPP expression than DARPP-32, as shown by stronger immunostaining with C-DARPP-32 than N-DARPP-32. The relevant IHC scores are depicted in boldface font. Click here for file
