# Supplementary figures and images for: t-DARPP regulates phosphatidylinositol-3-kinase-dependent cell growth in breast cancer
Source: Mol Cancer. 2010 Sep 13;9:240. doi: 10.1186/1476-4598-9-240 (PMC2945963; doi:10.1186/1476-4598-9-240)

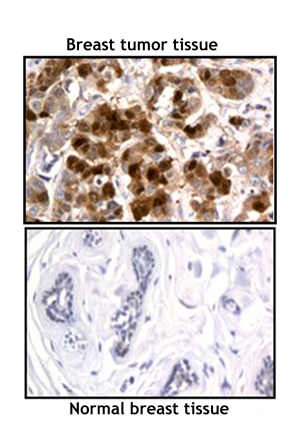

Supplement: Additional file 1 — Supplemental Figure S1. t-DARPP expression in primary breast tumors. A representative immunohistochemical staining for DARPP-32/t-DARPP of a breast tumor (upper panel) and a matched adjacent normal tissue (lower panel) samples from a tissue microarray with C-terminal DARPP-32 antibody. Note that the expression of DARPP-32/t-DARPP protein(s) is high in tumor tissue as opposed to very low to absent in adjacent normal tissue, as indicated by dark brown staining. [file 1476-4598-9-240-S1.TIFF]
