# Supplementary material for: t-DARPP regulates phosphatidylinositol-3-kinase-dependent cell growth in breast cancer
Source: Mol Cancer. 2010 Sep 13;9:240. doi: 10.1186/1476-4598-9-240 (PMC2945963; doi:10.1186/1476-4598-9-240)
Supplement: Additional file 2 — Supplemental Table S1. Summary of DARPP-32/t-DARPP immunostaining from breast tumor microarrays. DARPP-32/t-DARPP protein(s) expression was assessed by IHC staining on tissue microarrays containing 59 primary breast tumors and 25 adjacent normal breast tissue samples using C-terminal DARPP-32 antibody. Immunohistochemical results were evaluated for intensity and frequency of staining. The index score of staining was graded as 0 (negative), 1 (weak), 2 (moderate), and 3 (strong). The difference between DARPP-32/t-DARPP expression frequency and various parameters was assessed by Fisher Exact Test. IDC, invasive ductal carcinoma. [file 1476-4598-9-240-S2.DOC]

|  | **DARPP-32/t-DARPP index score** | | | |  |  | |
| --- | --- | --- | --- | --- | --- | --- | --- |
|  | | **0--1** | **2** | **3** | | |  |
| **Cases** | |  |  |  | | | p<0.01 |
| Normal | | 15 (60%) | 9 (36%) | 1 (4%) | | |  |
| Tumor | | 32 (54.2%) | 6 (10.1%) | 21 (35.5%) | | |  |
|  | |  |  |  | | |  |
| **Histology** | |  |  |  | | | p=0.008 |
| IDC | | 23 (47.9%) | 4 (8.3%) | 21 (43.7%) | | |  |
| Other | | 9(81.8%) | 2 (18.1%) | 0 | | |  |
|  | |  |  |  | | |  |
