# Supplementary material for: t-DARPP regulates phosphatidylinositol-3-kinase-dependent cell growth in breast cancer
Source: Mol Cancer. 2010 Sep 13;9:240. doi: 10.1186/1476-4598-9-240 (PMC2945963; doi:10.1186/1476-4598-9-240)
Supplement: Additional file 3 — Supplemental Table S2. Differential expression of DARPP-32 and t-DARPP in breast cancer tissues. IHC analysis of duplicate tissue microarray slides containing 19 primary breast tumor samples. Immunohistochemical staining was performed with N-terminal DARPP-32 antibody (EP720Y; abcam) (N-DARPP-32), which exclusively detects DARPP-32 protein, and C-terminal DARPP-32 antibody (Clone H-62; Santa Cruz Biotechnology) (C-DARPP-32), which detects both DARPP-32 and t-DARPP proteins. Both antibodies produced similar strong and specific staining in positive cases. The results indicated that 8 (42.1%) tumors exhibited relatively higher t-DARPP expression than DARPP-32, as shown by stronger immunostaining with C-DARPP-32 than N-DARPP-32. The relevant IHC scores are depicted in boldface font. [file 1476-4598-9-240-S3.RTF]

 	 IHC score	
Sample ID	N-DARPP-32	C-DARPP-32	
C1	0	0	
E1	0	0	
G1	0	4	
I1	12	12	
A2	6	9	
C2	0	0	
E2	0	0	
G2	6	9	
I2	12	12	
A3	0	0	
C3	0	1	
E3	2	4	
G3	0	0	
I3	6	6	
A4	0	0	
C4	0	0	
E4	0	0	
G4	0	2	
I4	9	12	
A5	12	12	
C5	0	0	
E5	0	4	
G5	0	0	
I5	0	0	
A6	12	12	
C6	12	8	
E6	0	0	
G6	12	12	
I6	0	0	
